# Urogenital Tuberculosis: A Narrative Review and recommendations for diagnosis and treatment

**DOI:** 10.1590/S1677-5538.IBJU.2024.0590

**Published:** 2025-01-20

**Authors:** André Avarese Figueiredo, José Carlos Truzzi, Augusto Azevedo Barreto, Eduardo Carvalho Siqueira, Marcos Lucon, Marcos Broglio, Karin Marise Jaeger Anzolch, Antônio Peixoto de Lucena Cunha, Bruno Vilalva Mestrinho, Leandro Koifman, José de Bessa, Luciano Alves Favorito

**Affiliations:** 1 Universidade Federal de Juiz de Fora Faculdade de Medicina Juiz de Fora MG Brasil Faculdade de Medicina da Universidade Federal de Juiz de Fora – UFJF – Juiz de Fora, MG, Brasil;; 2 Universidade Federal de São Paulo Escola Paulista de Medicina São Paulo SP Brasil Escola Paulista de Medicina da Universidade Federal de São Paulo - (EPM/UNIFESP), São Paulo, SP, Brasil; 3 Faculdade de Medicina Presidente Antônio Carlos Juiz de Fora MG Brasil Faculdade de Medicina Presidente Antônio Carlos, Juiz de Fora, MG, Brasil; 4 Universidade de São Paulo Faculdade de Medicina São Paulo SP Brasil Faculdade de Medicina da Universidade de São Paulo – FMUSP, São Paulo, SP, Brasil; 5 Santa Casa de São Paulo Disciplina de Urologia São Paulo SP Brasil Disciplina de Urologia da Santa Casa de São Paulo, São Paulo, SP, Brasil;; 6 Hospital Moinho de Vento de Porto Alegre Divisão de Urologia Porto Alegre RS Brasil Divisão de Urologia do Hospital Moinho de Vento de Porto Alegre, Porto Alegre, RS, Brasil; 7 Médicas de Minas Gerais Faculdade de Ciências Belo Horizonte MG Brasil Faculdade de Ciências Médicas de Minas Gerais, Belo Horizonte, MG, Brasil;; 8 Universidade Luziânia Faculdade de Medicina Luziânia GO Brasil Faculdade de Medicina da Universidade Luziânia, Luziânia, GO, Brasil; 9 Hospital Municipal Souza Aguiar Rio de Janeiro RJ Brasil Hospital Municipal Souza Aguiar, Rio de Janeiro, RJ, Brasil; 10 Universidade Estadual de Feira de Santana Faculdade de Medicina Feira de Santana BA Brasil Faculdade de Medicina da Universidade Estadual de Feira de Santana - UEFS, Feira de Santana, BA, Brasil; 11 Universidade do Estado do Rio de Janeiro Unidade de Pesquisa Urogenital Rio de Janeiro RJ Brasil Unidade de Pesquisa Urogenital, Universidade do Estado do Rio de Janeiro – UERJ, Rio de Janeiro, RJ, Brasil

**Keywords:** Tuberculosis, Tuberculosis, Urogenital, Review [Publication Type]

## Abstract

**Purpose:**

to review the more relevant aspects of urogenital tuberculosis (UGT) and make recommendations about the diagnosis and treatment.

**Materials and Methods:**

a literature review was conducted in the Pubmed, Embase and Scielo databases in search of studies on UGT in the past 60 years. A narrative review was performed concerning six topics of UGT diagnosis and treatment. Recommendations were made supported on degrees of evidence according to the modified GRADE system.

**Results:**

UGT suspicion occurs in persistent hematuria or pollakiuria with sterile pyuria; stenosis and/or thickening of the urinary tract; or chronic prostatitis or epididymitis. Urinary bacteriological tests have low sensitivity, and a negative test does not rule out UGT diagnosis. In ureteral stenosis, a double-J catheter or nephrostomy should be used early (up to 1 month) during pharmacological treatment and in single less than 2 cm stenosis endoscopic treatment may be attempted. Bladder augmentation with ileum, sigmoid or ileocecal segments should be performed when the contracted bladder capacity is less than 100 mL. Spontaneous voiding occurs in most patients after bladder augmentation.

**Conclusion:**

The diagnosis of UGT depends on a high degree of suspicion based on non-specific symptoms and radiological findings. Urinary bacteriological tests have low sensitivity, but even in the absence of diagnostic confirmation, treatment can be carried out through a combination of drugs for a period of six months. In the presence of ureteral stenosis or contracted bladder, complex but well stablished reconstruction procedures are necessary.

## INTRODUCTION

Tuberculosis is a transmissible disease that ranks among the top 10 leading causes of death worldwide and, with the exception of coronavirus disease 2019 (COVID-19), is the leading cause of death due to a single infectious agent. Brazil was one of 30 countries comprising 87% of new tuberculosis cases in 2022 (1). Extrapulmonary forms of tuberculosis account for 16% of tuberculosis cases (2), with urogenital tuberculosis (UGT) being the second most common presentation in some regions, ranking behind only the lymphatic disease type (3). UGT, like all forms of extrapulmonary tuberculosis, has common features, such as nontransmissibility and difficulty in diagnosis, owing to the nonspecificity of symptoms, the elimination of few bacilli in urine and difficult access for biopsy of the affected organs.

UGT can affect all urogenital organs and always occurs secondary to the hematogenous spread of pulmonary tuberculosis. Initially, the bacillus colonizes the kidney parenchymal region bilaterally; this step is followed by the development of granulomas in the region near the kidney glomeruli, in the loop of Henle, in the medullary region, close to the renal papilla. The initial lesions are bilateral and consist of granulomas without caseous necrosis or nodule formation and, therefore, without kidney radiological changes (4). These granulomas may remain latent throughout life; however, the disease may be reactivated mainly in medullary granulomas due to less vascularization and a greater chance of ischemia in this kidney area. Moreover, caseous necrosis, ulceration and erosion of the renal papillae to the urinary tract may occur, accompanied by bacilluria and descending dissemination of the infection. Thus, changes in the renal papilla characterize the initial radiological signs of renal tuberculosis (5). The disease spreads to the urinary system with the development of granulomas in the renal pelvis and ureter, causing thickening and obstruction, eventually reaching the bladder (6). The two narrowest sites of the urinary tract are the most commonly affected in the urinary system: the pyeloureteral junction and the ureterovesical junction (5).

The sequential evolution of urinary tuberculosis, from this reactivation of tuberculosis in the kidney to severe forms of the disease, has been well established in publications since the early 21th century (7, 8). An in-depth analysis of UGT case series revealed that the evolution of urinary tuberculosis follows a constant and progressive pattern: reactivation of tuberculosis occurs in one kidney (primary kidney), with the onset of clinically and radiologically detectable unilateral renal tuberculosis. As tuberculosis progresses through the urinary tract, the renal pelvis, ipsilateral ureter and bladder become involved. Most commonly, there is stenosis of the urinary tract with the possibility of loss of renal function due to obstruction, and the bladder may gradually contract; such contraction manifests as a low capacity and compliance bladder and causes vesicoureteral reflux to the contralateral kidney, which is secondarily affected. Thus, the primary kidney loses function because of the urinary tract obstruction caused by tuberculosis, and the secondary kidney may lose function owing to vesicoureteral reflux.

Male genital tuberculosis in some cases may be associated with renal tuberculosis. The prostate, according to autopsy studies (9), is the first organ of the male genital tract to be affected by hematogenous or urinary dissemination. Through the canalicular route, there is secondary involvement of the seminal vesicles, vas deferens and epididymis. In genital tuberculosis, the prostate is the most affected organ, but in most cases, prostate tuberculosis is asymptomatic. In contrast, epididymitis is the most frequent clinical manifestation, and tuberculosis of the epididymis can also occur alone via direct hematogenous dissemination (3). Male genital tuberculosis can manifest as six clinical syndromes: asymptomatic (simulating prostate cancer with PSA elevation and prostatic nodules); prostatic obstruction; chronic prostatitis; recurrent acute prostatitis; and prostatic abscess and chronic epididymitis, which may be unilateral or bilateral, with or without a cutaneous fistula (10).

Despite being a disease well known by urologists, UGT is still characterized by nonspecific symptoms, a lack of physician familiarity with its more specific clinical and radiological presentations, a low sensitivity of bacteriological tests and, consequently, a late diagnosis. Therefore, destruction of the urogenital tract may occur, with the appearance of renal exclusion, contracted bladder, renal failure and epididymal or prostate abscesses requiring complex reconstructive surgery.

In this review we will describe the more relevant aspects of urogenital tuberculosis and show some recommendations about the diagnosis and treatment of UGT.

## MATERIAL AND METHODS

In this study we carried out a review about urogenital tuberculosis. We analyzed papers published in the past 60 years in the databases of Pubmed, Embase and Scielo, found by using the key expressions: "Tuberculosis"; "Urogenital Tuberculosis"; "Tuberculosis treatment"; "Tuberculosis surgery"; "Prostate tuberculosis"; "Kidney Tuberculosis" and "Radiology". In this review we found several papers in these databases, and included only papers in English, and excluded case reports, editorials and opinions of specialists. Six topics of UGT diagnosis and treatment were elaborated by the Consultation Group of the Division of Infections from the Brazilian Society of Urology. From these topics, recommendations were made based on the *Grading of Recommendations Assessment, Development and Evaluation* (GRADE) system, according to guidance from the Ministry of Health. In the GRADE system, the level of evidence is classified as high, moderate, low or very low, and the strength of the recommendation is classified as strong or weak (11). After the recommendation, the following information is given (GRADE: level of evidence, strength of recommendation).

## RESULTS

### 1 - Clinical and radiological presentation.

Suspicion of UGT is based on the clinical/radiological situation at presentation. The three main symptoms of UGT are hematuria (35.6%), low back pain (34.4%) and pollakiuria (50.5%) (12). However, most patients present radiological kidney findings suggestive of UGT. In a review of almost 9,000 patients from 33 case series, in only 15% of the cases, there were no radiological changes in the kidneys (12). In another study evaluating the clinical/radiological presentation of 80 patients with UGT, it was observed that, in only 7.5% of the patients, no or minimal damage to the kidneys was observed, and in these patients, the only symptom present was hematuria. All the other patients presented with radiological changes (13). Male genital tuberculosis may be present without urinary tuberculosis and has specific clinical symptoms, mainly chronic prostate and epidydimal infections. Thus, the suspicion of a UGT should be based on three situations:

1. Clinical presentation: Persistent (more than three months) macroscopic hematuria or pollakiuria with sterile pyuria with no relevant findings in a urinary tract radiological investigation.
2. Radiological presentation: Regardless of the symptoms, the presence of the following radiological findings on computed tomography (CT) or magnetic resonance imaging (MRI) (14):
  Stenosis and/or thickening of the urinary tract with corresponding hydronephrosis, usually unilateral (bilateral stenosis is extremely rare).
  Stenosis may be intrarenal (infundibular), in the renal pelvis or ureter. Furthermore, the stenotic sites may be single or multiple, with hydronephrosis and corresponding total or partial loss of renal function. The evolution of the UGT may or may not be associated with a contracted bladder and contralateral hydronephrosis due to reflux.
3. Clinical presentation of the male genital tract:
  - Chronic prostatitis (pelvic pain or dysuria for more than 3 months, associated with lower urinary tract symptoms [LUTS]. The symptoms may be persistent or intermittent.)
  - Chronic epididymitis (unilateral or bilateral pain and thickening of the epididymis for more than 3 months) with or without cutaneous fistulization.

**Recommendations:**

1. UTG should be suspected in the following situations:
2. Persistent (more than three months) macroscopic hematuria or pollakiuria with sterile pyuria (GRADE: moderate, strong)
3. Stenosis and/or thickening of the urinary tract with corresponding hydronephrosis, usually unilateral on CT or MRI (GRADE: moderate, strong)
4. Chronic prostatitis or chronic epididymitis (GRADE: moderate, strong)

### 2 - Bacteriological investigation through urine culture and nucleic acid amplification (NAA) tests.

When UGT is suspected, bacteriological investigations in the urine are performed by specific culture for *Mycobacterium tuberculosis* or by identification of DNA fragments via nucleic acid amplification (NAA) techniques. The most studied NAA technique is the commercially available Xpert MTB/RIF, which also identifies resistance to rifampicin. Urine culture is considered the gold standard for diagnosis, but it takes six to eight weeks to obtain results, whereas it takes 24 to 48 hours to obtain results with NAA techniques. Both methods have high specificity. However, there is great uncertainty regarding the sensitivity values (3), and the high prevalence of false negatives makes diagnosis difficult; this situation is responsible for delays in treatment initiation. In addition, low-sensitivity diagnostic tests are unfeasible as the gold standard; therefore, other diagnostic strategies are needed.

The precise determination of the sensitivity and specificity of urine culture and NAA techniques requires studies that evaluate these tests in comparison with broader diagnostic strategies, a composite reference standard, including four different criteria of UGT diagnosis: 1) positive urine culture; 2) histological diagnosis; 3) radiological diagnosis; and 4) positive response to pharmacological treatment. There are four studies in the literature in which urine culture and NAA techniques were evaluated in relation to at least three of the four diagnostic criteria (15-18). The data from this analysis are described in Table-1. The total sensitivity of the culture was 40.1%, ranging from 24.0% to 56.4%. The total sensitivity of Xpert (NAA test) was 60.7%, ranging from 41.3% to 88.0%. When the two tests were used together, the total sensitivity was 63.1%, which was slightly greater than that of Xpert alone. The specificity of culture was 100% in all the studies, as it was the gold standard diagnostic method, and that of Xpert was 99.4%.

**Table 1:** The table shows the data description of four articles analyzed in this review (15-18) with analysis of the sensitivity and specificity of urine culture and GeneXpert MTB in relation to a composite reference standard.

Author		Pang, et al. (2017) (15)	Samuel, et al. (2018) (16)	Chen, et al. (2019) (17)	Liu, et al. (2021) (18)	Total
N		163	100	302	112	677
TB cases		81	55	150	83	369
No TB cases		82	45	152	29	308
Culture medium		LJ	MGIT 960	MGIT 960	LJ + MGIT 960	
**CRS**						
	Bacteriological	X	X	X	X	
	Histology	X	X	X	X	
	Radiological		X	X	X	
	Pharmacological response	X		X	X	
**SENSITIVITY**						
	Culture	45.7%	56.4%	24.0%	53.0%	40.1%
	GeneXpert MTB	63.0%	69.1%	41.3%	88.0%	60.7%
	Both	65.4%	72.7%	42.67%	91.6%	63.1%
**SPECIFICITY**						
	Culture	100%	100%	100%	100%	100%
	GeneXpert MTB	98.8%	100%	100%	96.6%	99.4%

N = number of patients; TB = tuberculosis; LJ = Lowenstein–Jensen solid medium; MGIT 960 = liquid culture systems BACTEC MGIT 960; CRS = composite reference standard.

In the 2021 Guideline, the World Health Organization recommended that in patients with signs and symptoms of extrapulmonary tuberculosis (including UGT), Xpert MTB/RIF should be the initial diagnostic test performed (19).

**Recommendations:**

1. The investigation of UGT in suspected cases should be performed initially and preferably with the Xpert MTB/RIF test in urine (GRADE: moderate, strong).
2. Urine culture for *Mycobacterium tuberculosis* must be performed in at least three samples on different days and must be performed together with the Xpert MTB test, since culture alone has a sensitivity of only 40%, and both tests have a sensitivity of 63% (GRADE: moderate, strong).
3. A diagnosis of active UGT is made on the basis of a positive urine culture or Xpert MTB result, as both tests have a specificity of almost 100% (GRADE: moderate, strong).
4. Negative results for culture or Xpert MTB in urine do not rule out the diagnosis of UGT, as these methods have low sensitivity. This situation implies that there is a need for other criteria for the diagnosis of UGT (GRADE: moderate, strong).

### 3 - Clinical, laboratory and radiological criteria for UGT diagnosis in negative bacteriological investigation cases.

Due to the low sensitivity of bacteriological tests (culture and NAA techniques), there is a need for clinical, laboratory and radiological criteria for UGT diagnosis in negative bacteriological investigation cases, which would allow pharmacological treatment to be initiated. Currently, there are no accepted diagnostic criteria for UGT; therefore, the initiation of treatment is based on experience and common sense. The lack of standardized diagnostic criteria leads to great variability in the diagnosis of UGT. The authors of this review decided, out of necessity, to propose provisional diagnostic criteria until studies could validate them.

1. Definitive diagnosis:
  - Positive result of culture or NAA test for Mycobacterium tuberculosis in urine; sperm; renal, prostatic or epididymal abscess; or renal, bladder, prostate or epididymal biopsy sample.
  - Presence of a granuloma, with or without caseous necrosis, in a biopsy sample of an organ/tissue of the urogenital tract.
2. Probable diagnosis:
  Evidence of previous tuberculosis, namely, a history or radiological signs of pulmonary or extrapulmonary tuberculosis, a positive interferon gamma release assay (IGRA) result, or a positive purified protein derivative (PPD) test (reaction greater than 5 mm), associated with the following:
  - Persistent macroscopic hematuria (more than 3 months) without radiological changes in the urinary tract on CT or MRI.
  - Thickening and/or stenosis of the urinary tract with or without a nonfunctioning kidney for no apparent reason.
  - Contracted bladder: Bladder capacity less than 100 mL and radiological observance of a small bladder that has thickened walls without diverticula and is associated with at least one kidney with hydronephrosis owing to thickening and/or stenosis of the urinary tract.
  - Chronic epididymitis (more than 3 months) without improvement with the use of conventional antibiotic therapy with or without epididymal-cutaneous fistula.
  - Chronic prostatitis (more than 3 months) without improvement with the use of conventional antibiotic therapy.
3. Possible diagnosis:

The same findings as for the probable diagnosis, but with no evidence of previous tuberculosis.

Pharmacological treatment should be initiated for patients with a definitive or probable diagnosis, whereas for patients with a possible diagnosis, pharmacological treatment should be initiated after consensus between the medical team and the patient, with an explanation of the risks and benefits.

**Recommendations:**

Currently, without specific criteria validated in the literature, in the absence of bacteriological or histological confirmation, the initiation of treatment for UGT should be based on the presence of suggestive clinical situations or radiological findings (GRADE: very low, weak).

### 4 - Pharmacologically treatment.

The tuberculosis treatment regimen should be administered according to the recommendations of the World Health Organization and comprises two phases: intensive (or attack) and maintenance (20). The aim of the intensive phase is to rapidly reduce the bacillary population and eliminate bacilli with natural resistance to any of the drugs. The aim of the maintenance phase is to eliminate latent or persistent bacilli and reduce the possibility of disease recurrence. In Brazil, the basic regimen for tuberculosis treatment consists of four drugs in the intensive phase (rifampicin, isoniazid, pyrazinamide and ethambutol), which lasts 2 months, and two drugs (rifampicin and isoniazid, which have greater bactericidal power) in the maintenance phase, which lasts 4 months (20). The main concern during treatment is the hepatic toxicity of the drugs.

Liver disease should be managed with caution, and the use of alternative drugs may be necessary. Patients who drink alcohol should be instructed to discontinue alcohol intake because of the risk of drug-induced hepatitis. At the beginning of treatment, the following tests should be performed: blood glucose and liver and kidney function tests. During treatment, these tests should be repeated at the clinician's discretion.

**Recommendations** (20):

The treatment of UGT comprises two phases (Table-2): an intensive (or attack) phase with rifampicin, isoniazid, pyrazinamide and ethambutol that lasts for two months and a maintenance phase with rifampicin and isoniazid that lasts for months (GRADE: high, strong).

**Table 2:** The tuberculosis treatment regimen according to the recommendations of the World Health Organization (20).

Scheme	Weight range	Unit/dose	Duration
RHZE 150/75/400/275 mg	20 to 35 kg	2 tablets	2 months (intensive phase)
	36 to 50 kg	3 tablets	
	51 to 70 kg	4 tablets	
	Above 70 kg	5 tablets	
HR 300/150 mg or 150/75 mg	20 to 35 kg	1 tablet 300/150 mg or 2 tablets 150/75 mg	4 months (maintenance phase)
	36 to 50 kg	1 tablet 300/150 mg + 1 tablet 150/75 mg or 3 tablets 150/75 mg	
	51 to 70 kg	2 tablets 300/150 mg or 4 tablets 150/75 mg	
	Above 70 kg	2 tablets 300/150 mg + 1 tablet 150/75 mg or 5 tablets 150/75 mg	

R-rifampicin; H-isoniazid; Z-pyrazinamide; E-ethambutol

### 5 - Reconstructive surgery in stenosis of the urinary tract.

Tuberculosis causes stenosis of the entire urinary tract and may be intrarenal (infundibular or renal pelvis) or ureteral, single or multiple; the most frequent site is the distal ureter. Urinary tract stenosis is the main cause of loss of renal function in patients with tuberculosis and almost always occurs unilaterally (3). In two retrospective case series of renal tuberculosis, the following factors were associated with a greater chance of preserving renal function at the initial diagnosis: distal ureteral stenosis, good cortical thickness and a glomerular filtration rate greater than 15 mL/min. (21, 22).

After the initiation of pharmacological treatment, ureteral obstruction may worsen with the loss of renal function due to fibrosis progression. In a retrospective study of 77 patients with ureteral stenosis, early placement (less than 1 month) of a double J catheter or nephrostomy reduced the chance of nephrectomy by 50% and may have led to the resolution of the stenosis in some cases (23). The definition of reconstructive surgery varies according to the segment and extent of the ureter involved, the ipsilateral renal function and the degree of bladder involvement. Definitive surgical treatment can be performed four to six weeks after pharmacological treatment is initiated (24).

For cases of stenosis smaller than two centimeters, in which it is possible to pass a guidewire, endoscopic treatment with balloon dilation and a ureteral incision can be attempted. Balloon dilation can be performed in a retrograde or anterograde manner, with a double J catheterization for 6 weeks. In 1982, Murphy et al. reported a success rate of 64%, with a mean of 4 dilations per patient (25). In 2005, Sinha et al. reported a 59% success rate at 12 months (26). Endoureterotomy is performed under direct vision with a cold knife, electrode or laser, and the incision should be made anteromedially in the distal ureter and posterolaterally in the proximal ureter until the periureteral fat is visualized (24).

In the event of endoscopic treatment failure or strictures greater than two centimeters, traditional open, laparoscopic or robotic reconstructive surgery should be performed (24). The techniques used are the same as those employed for other forms of ureteral stricture: short stricture, usually in the middle or upper ureter, primary anastomosis may be sufficient; upper ureteral stenosis can be surgically treated with ureteropyelostomy if there is short stenosis with the extrarenal pelvis; or with ureterocalicostomy when the renal pelvis is not greatly dilated but there is calyceal dilatation. When the distal ureter is affected, ureteral reimplantation can be performed, possibly with a psoas hitch or Boari flap (27, 28). In the middle segments, transuretero-anastomosis may be necessary, and if the stenosis is throughout the ureter, replacement by a segment of the ileum (ileal ureter) or autotransplantation may be necessary (24, 25). If joint bladder augmentation is needed, the ileocecal segment is preferred, with the cecum used for bladder augmentation and the ileum used for reconstruction of the ureter (8, 29).

**Recommendations:**

- In the presence of ureteral stenosis due to tuberculosis, a double-J catheter or nephrostomy should be used early (up to 1 month), before the beginning of pharmacological treatment, in cases in which kidney function preservation is necessary (GRADE: moderate, strong).
- For patients with a single stenotic site measuring less than 2 cm through which it is possible to pass a guidewire, endoscopic treatment with balloon dilation or endoureterotomy followed by the insertion of a double-J catheter for 6 weeks can be attempted (success rate of up to 60%) (GRADE: low, weak).
- For cases of complex strictures (those with multiple strictures greater than 2 cm in size or the impossibility of passing through a guidewire) or failure of endoscopic treatment, traditional open, laparoscopic or robotic reconstructive surgery should be performed (GRADE: low, strong).

### 6 - Surgical treatment for contracted bladder.

Bladder tuberculosis is always secondary to renal tuberculosis that has spread through descending urinary dissemination of the infection. In more advanced bladder tuberculosis, the detrusor muscles are replaced by fibrotic tissue, resulting in a contracted bladder (8). Radiologically, there is diffuse bladder wall thickening without trabeculations or diverticula, and functionally, there is pollakiuria with a voiding interval of less than 1 hour and a bladder capacity of less than 100 mL. Commonly, UGT is associated with uni- or bilateral secondary vesicoureteral reflux (8).

Between 1969 and 2014, 11 case series of patients with contracted bladder due to tuberculosis were published (8, 30-39). Among the 316 patients, 64% were men, and their mean age was between 30 and 40 years. Bladder augmentation was performed for 90% of the patients, and cystectomy with an orthotopic neobladder was performed for 10%. Bladder augmentation was performed with the ileal segment for 35.4% of the patients, with detubularization of the ileum in all patients, except a few patients from two older series published in 1969 and 1970. The sigmoid was used for 38.9% of the patients, with detubularization in almost all patients, and the ileocecal segment was used for 25.8% of patients, but without detubularization, in its original configuration. In patients who underwent neobladder surgery, the Studer technique with the ileum was the most commonly employed (73.3%), but other segments were also used (sigmoid and ileocecum). The success criteria varied between case sets but were usually defined as voiding improvement with an increasing voiding interval and preservation of the upper urinary tract. Good voiding results were achieved for 80 to 100% of the patients. However, there were patients whose condition progressed to end-stage renal failure in some case series (8, 30, 35, 37, 39). Despite the improvement in voiding with good reservoir quality, there was progression of kidney injury regardless of the success of surgery and improvement of the bladder reservoir. The vast majority of patients spontaneously urinated without the need for self-catheterization after surgery, which initially occurred in 85.8% of the patients; however, this value reached 94.2% after new surgery for bladder outlet obstruction, such as transurethral resection of the prostate. In two case series (8, 33), data from the urodynamic evaluation after bladder augmentation were available. In cystometry, the presence of detrusor overactivity (or its equivalent in the augmented bladder, where the elevation of intravesical pressure can occur by contraction of the bladder or intestinal segment) occurred in 72% of the patients and was not associated with low capacity (8). Phasic contractions, apparently of the intestinal segment triggered by bladder filling, were observed. In the pressure-flow study, all patients urinated because of a voluntary increase in abdominal pressure (Valsalva maneuver), which, in some patients, may have occurred in association with increased bladder pressure owing to contraction of the augmented bladder. Worse results of bladder augmentation were associated with low reservoir capacity but not with increased bladder contraction (8).

The largest case series of patients with UGT was published in 1997 and was derived from experience in Moscow since 1960, with the description of 4298 patients and surgical treatment of 2364 (55%) patients (40). A contracted bladder was present in 454 patients. Owing to the lack of detailed case descriptions, these cases were not included in the present review. However, because of the magnitude of experience, conclusions regarding surgical treatment techniques for contracted bladders have become relevant. In almost all cases, the sigmoid was used (95.6%), with the justification that in ileal augmentation, there is greater postvoiding residue due to the lower contractility of the ileal segment than the sigmoid. The use of cystoprostatectomy and an orthotopic neobladder comprising an ileocecal segment with ureteral anastomosis to the terminal ileum, bladder augmentation with cecum and invagination of the appendix stump into the urethra was proposed in men with greater bladder and prostatic destruction by tuberculosis to avoid stenosis of the enterovesical anastomosis with a very contracted bladder and to allow the removal of all scar tissue. In men, the optimal choice between bladder augmentation versus cystoprostatectomy and orthotopic neobladder is not well established, but a neobladder may be considered in patients with greater bladder and prostatic lesions with very small bladders (capacity less than 20 mL) and the presence of pelvic pain (36, 38, 40).

In only one retrospective and nonrandomized study was there a comparison between the techniques used for bladder augmentation (8). In this study, good results (voiding interval greater than or equal to two hours) were associated with the use of an ileocecal segment without detubularization and a sigmoid segment with detubularization. The use of sigmoid without detubularization as well as the presence of pelvic pain (a sign of tuberculous prostatitis) were associated with poor results.

Ureteral reimplantation should be performed in cases of stricture, but it is not necessary in cases of reflux (38). The presence of some degree of renal failure is not a contraindication to bladder enlargement, as it allows for a better quality of life due to improved voiding, and patients with an augmented bladder can appropriately receive a kidney transplant (8).

**Recommendations:**

1. In patients with bladder tuberculosis, bladder augmentation with an intestinal segment is indicated when the bladder capacity is less than 100 mL (GRADE: moderate, strong).
2. In patients with very small bladders (capacity less than 20 mL) or in those with UGT associated with pelvic pain, cystoprostatectomy and an orthotopic neobladder may be considered (GRADE: very low, weak).
3. When bladder augmentation or orthotopic neobladder surgery is performed, the ileum, sigmoid and ileocecal segments can be used. Detubularization and reconfiguration of the intestinal segment should be performed, but the ileocecal segment can be used in its original form without detubularization (GRADE: low, weak).
4. Ureteral reimplantation is indicated in patients with ureteral stricture but may not be performed in patients with reflux (GRADE: low, strong).
5. Spontaneous voiding occurs in most patients after bladder augmentation. In patients with a large volume of postvoiding residual urine, bladder outlet surgery, such as transurethral resection of the prostate or urethral surgery, should be performed to avoid self-catheterization (GRADE: very low, weak).

## CONCLUSIONS

The diagnosis of UGT depends on a high degree of suspicion based on non-specific symptoms and radiological findings. Urinary bacteriological tests have low sensitivity, but even in the absence of diagnostic confirmation, treatment can be carried out through a combination of drugs for a period of six months. In the presence of ureteral stenosis or contracted bladder, complex but well stablished reconstruction procedures are necessary. A better knowledge of UGT features is essential to improve diagnosis and treatment management.
